# An organizing framework for informal caregiver interventions: detailing caregiving activities and caregiver and care recipient outcomes to optimize evaluation efforts

**DOI:** 10.1186/1471-2318-11-77

**Published:** 2011-11-22

**Authors:** Courtney Harold Van Houtven, Corrine I Voils, Morris Weinberger

**Affiliations:** 1 Center for Excellence in Health Services Research and Development in Primary Care, Durham Veterans Affairs Medical Center, NC, USA; 2 Department of Medicine, Duke University Medical Center USA, Durham, 27710, NC, USA; 3 Department of Health Policy and Administration, University of North Carolina at Chapel Hill, NC, USA

## Abstract

**Background:**

Caregiver interventions may help improve the quality of informal care. Yet the lack of a systematic framework specifying the targets and outcomes of caregiver interventions hampers our ability to understand what has been studied, to evaluate existing programs, and to inform the design of future programs. Our goal was to develop an organizing framework detailing the components of the caregiving activities and the caregiver and care recipient outcomes that should be affected by an intervention. In so doing, we characterize what has been measured in the published literature to date and what should be measured in future studies to enable comparisons across interventions and across time.

**Methods:**

Our data set comprises 121 reports of caregiver interventions conducted in the United States and published between 2000 and 2009. We extracted information on variables that have been examined as primary and secondary outcomes. These variables were grouped into categories, which then informed the organizing framework. We calculated the frequency with which the interventions examined each framework component to identify areas about which we have the most knowledge and under-studied areas that deserve attention in future research.

**Results:**

The framework stipulates that caregiver interventions seek to change caregiving activities, which in turn affect caregiver and care recipient outcomes. The most frequently assessed variables have been caregiver psychological outcomes (especially depression and burden) and care recipient physical and health care use outcomes.

**Conclusions:**

Based on the organizing framework, we make three key recommendations to guide interventions and inform research and policy. First, all intervention studies should assess quality and/or quantity of caregiving activities to help understand to what extent and how well the intervention worked. Second, intervention studies should assess a broad range of caregiver and care recipient outcomes, including considering whether expanding to economic status and health care use of the caregiver can be accommodated, to ease subsequent economic evaluations of caregiving. Third, intervention studies should measure a common set of outcomes to facilitate cross-time and cross-study comparisons of effectiveness.

## Background

The provision of informal care, defined as unpaid custodial or medical care to family members or friends who have experienced a loss in independence, is common worldwide. Despite an increasing reliance on paid long-term care for elders, informal care persists as the most common form of long-term care provided in the U.S. In 2009, 45 million households had provided informal care to a relative or friend aged 50 or older in the past year [1]. Given the projected rapid growth of elderly adults, especially those aged 85 and older, informal caregiving will continue to be critical in the foreseeable future.

Programs to support informal caregivers have the potential to improve outcomes of both caregivers and care recipients. Many caregiver programs have been evaluated as part of research protocols; others are implemented by diverse agencies without formal assessment; and still others are mandated without standardization, such as the National Family Caregiver Support Program, Older Americans Act, Title II E, 2000. Efforts to evaluate programs have been hampered by the lack of an organizing framework for their design and assessment. This, in turn, has impeded the ability to identify "best practices" as we seek to prepare caregivers for the challenges they face and to maximize outcomes of caregivers and recipients. Standardization of caregiver programs or policies may be difficult because they are mandated at national, state, or local levels, or are often not developed with evaluation in mind. In contrast, research-based interventions, which facilitate harmonization of approaches across key domains such as the outcomes evaluated and reported, are developed based on reports on what was effective in past interventions. By interventions, we include broad strategies that offer diverse programs (e.g., social support, education, tangible resources) offered by diverse entities (e.g., health care system, government, social agencies).

Whereas researchers will often choose to tailor their own interventions based on theory, conceptual models, results of past intervention studies, and knowledge about complex intervention design approaches [2], there is little guidance on how to either structure the inputs of caregiving programs or select measures that optimize evaluation efforts. Evaluating measures of inputs into the caregiving process (hereafter called "caregiving activities", that is, all behavior or tasks that are performed as a requirement of being a caregiver of a disabled or dependent adult) helps provide signals about the specific mechanisms that were affected by an intervention, which can be helpful when outcomes do not change. Furthermore, providing more detail on the caregiving activities and outcomes will improve the ability to make cross-study comparisons of interventions.

**Figure 1:**
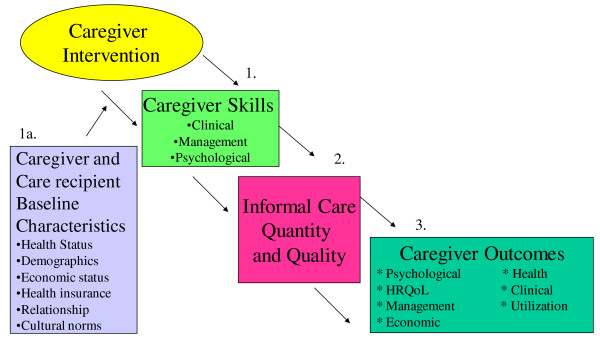
**Original Draft of Organizational Framework**.

To address these needs, we developed an organizing framework detailing the expected paths by which caregiver interventions affect caregiver activities and, in turn, key caregiver and care recipient outcomes. This initial framework was based on the literature, our experience in caregiving research, and comments from several clinical and research caregiving experts regarding the initial version of the framework (The initial framework appears as Figure 1 and all persons from whom we sought input are listed in the acknowledgements section). We then reviewed studies published during the past decade that evaluated caregiver interventions, classifying which, and to what extent, outcomes have been measured. This led to refinement of the framework and recommendations for assessing outcomes in future studies to enable comparisons to be made across interventions and across time.

## Methods

### Data Set

We used systematic review techniques to conduct our literature search. Specifically, we searched Medline/Pubmed, Psycinfo and Cinahl using ("informal care*" or "caregiv*" or "carer") and (program or trial or intervention). We limited the Psycinfo search to intervention studies, which is a search criterion not available as a filter in the other databases, and we limited the Cinahl search to articles not appearing in Medline, since those would be duplicative with the Medline/Pubmed search. We restricted our search to studies conducted in the U.S. because cultural and policy differences to support informal caregivers of the elderly make cross-national comparisons challenging; for a systematic review of international trials, see Thompson and Spillsbury, 2007. We restricted our search to reports of studies published between January 1, 2000 and August 31, 2009, a period during which the preeminent trials in the U.S. were conducted and published. The inclusion criteria were: (1) included caregivers of adults ("All adult (18 plus years)" was the Medline limit, and "All adults 18 plus" was the Psycinfo limit); (2) included an intervention (either randomized or single-arm, pre-post design); (3) reported quantitative outcomes that came directly from subjects (not perceptions of change reported by third parties); and (4) care recipients resided in the community. We did not exclude pilot studies or feasibility studies. When there were multiple publications from the same study, we counted activities and outcomes only once per study. Because our goal was to determine to what extent outcomes have been assessed rather than to assess the effectiveness of interventions, we neither evaluated the quality of each article nor whether the outcomes were significantly affected by the intervention, nor did we limit our search to specific disease conditions. The interventions varied in content from in-person psycho-educational counseling to tele-support to in-home individual training, thus, in order to truly profile differences in intervention doses, conceptual models, one would need to return to the source articles for more information.

### Measurement of Outcomes

For each study, we recorded all primary and secondary outcomes. In so doing, we noticed that: (1) authors used different names for the same constructs (e.g., mood and well-being to assess psychological morbidity); (2) different measures were used for the same construct (e.g., PRIME-MD vs. CES-D to assess depression); and (3) some constructs lacked standardized definitions (e.g., "objective burden" and "subjective burden" measured with similar instruments). Therefore, we developed a coding scheme comprising an exhaustive list of constructs in an iterative manner, adding new constructs as we encountered them. For all articles, the outcome variables were classified according to the final coding scheme.

Two investigators (CHV and CIV) first classified all caregiver outcomes and care recipient outcomes separately and counted the number of articles for each caregiver and care recipient outcome. We then grouped the individual constructs into higher order categories (e.g., nursing home use and hospital use were compiled into the category "care recipient health care utilization"). We continued to compare our initial draft of the framework to the categories identified in the structured literature review in this manner and then revised the framework based on the results of the review. We sought feedback about the framework from clinician and non-clinician caregiving experts to ensure that the revised framework portrayed mechanisms by which caregiving interventions affect caregiver and care recipient outcomes. The result was that the initial framework was less detailed than the final framework in how we classified caregiving activities (copies of the original framework available appears as Figure A1 in the Appendix).

## Results

### Results of the Structured Literature Review

**Figure 2:**
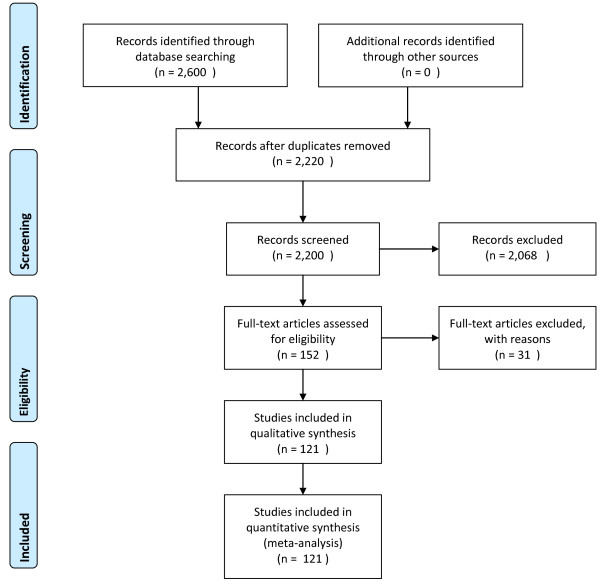
**PRISMA 2009 Flow Diagram of the Structured Literature Review^i^**.

The search strategy produced 2, 600 reports (2, 224 in Pubmed/Medline; 150 in Psycinfo; and 226 in Cinahl), of which to 2, 220 were unique reports (Figure 2). We used the ancestry approach to identify additional articles. Based upon review of the abstracts for relevance, we excluded 2, 068 reports. After reading the methods and results sections of the 152 remaining reports, we excluded 31 reports that did not meet our inclusion criteria: 18 lacked quantitative outcomes; 4 were case studies; 2 were non-U.S.; 2 were not caregiver studies (paid home health or nurses were the focus); 1 had outcomes reported from third parties; 1 had minors as care recipients; 1 was an observational study; 1 was in a nursing home; and 1 had outcomes that had already been counted in another identified article. Thus, 121 individual reports were included in our analysis. The full list of references for these studies appears in Additional File 1.

### Major Components of the Organizing Framework

**Figure 3:**
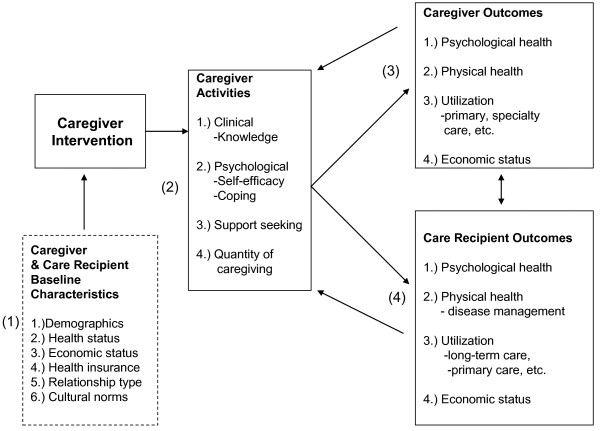
**Organizing Framework for Caregiver Interventions**.

The major components of our framework (Figure 3) include: (1) caregiver and care recipient baseline characteristics (demographic characteristics, health status, economic status, health insurance, relationship type, and cultural norms); (2) caregiving activities (clinical skills and knowledge, psychological skills, support seeking, and quantity of caregiving); (3) caregiver outcomes (psychological health, physical health, health care utilization such as primary or specialty physician care, and economic status); and (4) care recipient outcomes (disease management tasks, psychological health, physical health, health care utilization such as community-based or institutional long-term care, respite care, or primary physician care, and economic status). Caregiver activities are distinct from outcomes because they relate to the tasks that caregivers perform (caregiving input). For example, support seeking skills entail how well the caregiver can garner social support for herself as a caregiver, but the ultimate outcome related to this skill activity would be decreased caregiver burden or caregiver depression. Notably, caregiver and care recipient outcomes affect each other (double-headed arrows), and caregiver and care recipient outcomes feedback to caregiving activities. Each framework component is discussed in detail below. All data in this review, including spreadsheets, excluded studies, and search results, are available upon request.

### Caregiver and Care Recipient Baseline Characteristics

Our organizing framework explicitly accounts for caregiver and care recipient baseline characteristics, which affect the design of an intervention, the uptake of an intervention and or response to an intervention. For example, caregivers' health, wealth, and cultural norms, and care recipients' education [3] may affect adherence to intervention [4], caregiving activities, and care recipient outcomes. Additionally, the disease or condition of the care recipient is important because it determines the type of caregiver support and/or training needed and because it can provide information about the likely duration of the caregiving episode, which can affect important caregiver and care recipient outcomes.

### Caregiving Activities

Caregiving activities encompass: (1) clinical skills and knowledge; (2) psychological skills and resources; (3) support seeking skills; and (4) quantity of caregiving. Our classification of some of these measures as "caregiving activities" overrode the authors' description of these constructs as outcomes in the literature review. For example, whereas our initial framework considered the main two caregiving activities to comprise of quantity and quality of informal care, we realized through the review that the caregiving input was comprised of many more types of caregiving activities. Therefore, we adjusted the framework. The activities as conceptualized here, allow for a more immediate measure of an intervention's effects than more distal outcomes.

#### Clinical Skills and Knowledge

Informal caregivers often are required to provide medical and/or custodial care, including assistance with activities of daily living (ADLs) and instrumental activities of daily living (IADLs). The required skills can include medication management, help with transferring, and wound care or changing an intravenous line. Making decisions or solving problems on behalf of the patient when necessary are also considered clinical skills. Another related skill is knowledge, such as knowledge about services available for the caregiver or recipient or knowledge about how to exercise. Knowledge is important for carrying out necessary tasks and is an important prerequisite for behavior change.

#### Psychological Skills and Resources

Informal caregivers often experience significant stress and mental health problems--including depression and caregiver burden--that may be mitigated through attention to psychological skills and resources. Coping skills help caregivers come to terms with their situation using psychological and practical strategies [5], including constructing a larger sense of the illness, praying for strength to keep going, reducing expectations, and reminding oneself that a care recipient's decline can be expected with aging [6]. Self-efficacy, which includes task mastery, refers to confidence that one can perform a specified behavior. Enhancing coping and self-efficacy may minimize the negative emotional strain of caregiving and/or maximize the positive aspects of caregiving.

#### Support Seeking Skills

As case managers, gate keepers, and first-line providers, caregivers must develop organizational, tactical, and recruiting skills that help support their efforts. Organizational skills facilitate tasks such as scheduling medication and ensuring that needed medical care is received. Tactical skills allow caregivers to anticipate what kind of help is needed and what help is going to be needed next, including how to get that help. In terms of recruiting skills, caregivers must know: 1) what outside resources are available and whether these resources are covered by the recipients' insurance benefits; 2) how to screen, secure, and manage paid caregivers; 3) when to involve other informal caregivers; and 4) how to coordinate multiple providers and community resources.

#### Quantity of Caregiving

Quantity of informal care provided, also known as objective burden, refers to the time spent caregiving, which is vital to understanding the opportunity cost of a caregiver's time. These data are critical to examine cost-effectiveness of an intervention.

### Caregiver Outcomes

Evaluating the effect of caregiver interventions is complex because there can be both positive outcomes of caregiving (e.g., role satisfaction, improved relationships) and negative outcomes (e.g., caregiver depression, anxiety, stress, burden). We classified caregiver outcomes into four categories: psychological health; physical health; utilization; and economic status.

### Care Recipient Outcomes

Outcomes of care recipients are similar to those of caregivers but also include disease management skills. Disease management skills include care recipients' efforts to care for themselves given their disease profile (e.g. exercise for post-MI patients). Psychological health is categorized into nonsocial (e.g., depression) and social (e.g., family functioning) components. Physical health includes symptoms and sequelae of disease or illness. Finally, care recipient health care utilization and economic status are separate categories.

### The Extent to which the Framework Components Have Been Examined

Characteristics of the 121 studies in our analysis are shown in Table 1, including care recipient disease type, whether it was theory-based, and a checklist of types of measures included across caregiver activities caregiver outcomes, and care recipient outcomes. We noted a few general observations about the studies. Although the majority (66.1%) targeted caregivers of patients with Alzheimer's disease or dementia, care recipients had a diverse set of conditions, including cancer (11.6%), general frailty, multi-morbidity or disability (7.4%), post-stroke (4.1%), and other conditions (10.8%). Fifty-five (45%) of the studies examined caregiver activities as outcomes; 107 (88.4%) considered caregiver outcomes; and 62 (51%) reported care recipient outcomes. About 42% of the articles explicitly mentioned a conceptual model or theory on which the intervention was based, the most frequent of which were Pearlin's stress process model [7], Lazarus's transactional theory of stress [8], or Bandura's social learning/social cognitive theory [9]. Notably, some articles mentioned theories but did not explain how the theory was used to develop or adapt interventions for their studies. The following sections report the most frequent activities and outcomes examined in these reports.

**Table 1:** Studies included in the literature review, and checklist of features and measures

Authors	Publication Year	Disease/Condition	Model/Theory	Caregiving Activities (ACT)	Caregiver Outcomes? (CG)	Recipient Outcomes? (CR)	All Three?	**ACT/CG/CR**^**1 **^**Outcomes?**
Akkerman, Ostwald et al.	2004	Dementia	√		√			CG
Albert, Im et al.	2002	TBI	√	√	√			ACT/CG
Bakas, Farran et al	2009	Stroke	√		√	√		CG/CR
Bank, Arguelles et al	2006	Dementia		√	√			ACT/CG
Beauchamp Irvine et al	2005	Dementia	√	√	√			ACT/CG
Belle, Burgio et al	2006	Dementia		√	√			ACT/CG
Boerner, Schulz et al	2004	Alzheimer's	√		√			CG
Bourgeois, Schulz et al	2002	Dementia		√	√			ACT/CG
Burgio, Stevens et al	2003	Dementia		√	√			ACT/CG
Burns, Nichols et al	2003	Dementia			√			CG
Callahan, Boustani et al	2006	Alzheimer's	√		√	√		CG/CR
Carneval, Anselmi et al	2002	Traumatic Brain Injury			√			CG
Carter	2006	Cancer			√			CG
Castro, Wilcox et al	2002	Dementia	√	√	√			ACT/CG
Chee, Gitlin et al	2007	Alzheimer's			√			CG
Clark, Lester et al	2000	Multimorbidity	√	√	√			ACT/CG
Clark, Rummans, et al	2006	Cancer			√			CG
Connell, Janevic et al	2009	Dementia	√	√	√			ACT/CG
Coon, Thompson et al	2003	Dementia	√		√			CG
Corcoran, Gitlin et al	2001	Dementia			√			CG
Dellasega, Zerbe et al	2002	Frailty		√	√	√	yes	ACT/CG/CR
Devor, Renvall et al	2008	Dementia		√	√			ACT/CG
Dew, Goycoolea et al	2004	Heart	√		√			CG
Drentea, Clay et al	2006	Alzheimer's		√	√			ACT/CG
Eisdorfer, Czaja, et al	2003	Dementia		√	√			ACT/CG
Elliott, Brossart et al	2009	Spinal cord	√		√			CG
Elliott, Berry et al	2008	Spinal cord	√	√	√			ACT/CG
Farran, Gilley et al	2004	Dementia			√			CG
Farran, Gilley et al	2007	Dementia	√	√	√	√	yes	ACT/CG/CR
Farran, Stafileno et al	2008	Dementia	√		√			CG
Finkel, Schulz et al	2007	Dementia		√	√			ACT/CG
Fortinski, Kulldorff et al	2009	Dementia	√		√			CG
Gallagher-Thompson, Coon et al	2003	Dementia		√		√		ACT/CR
Gallagher-Thompson, Gray et al	2007	Alzheimer's	√		√	√		CG/CR
Gallagher-Thompson, Lovett et al	2000	Dementia	√	√	√	√	yes	ACT/CG/CR
Gant, Steffen et al	2007	Dementia		√	√	√	yes	ACT/CG/CR
Garand, Buckwalter et al	2002	Dementia	√		√			CG
Gaugler, Roth et al	2008	Alzheimer's			√			CG
Gerdner, Buckwalter et al	2002	Dementia	√			√		CR
Gitlin, Burgio et al	2003	Alzheimer's	√		√	√		CG/CR
Gitlin, Corcoran et al	2001	Alzheimer's	√	√		√		ACT/CR
Gitlin, Hauck et al	2005	Alzheimer's		√	√	√	yes	ACT/CG/CR
Gitlin, Hauck et al	2006	Dementia	√	√	√	√	yes	ACT/CG/CR
Gitlin, Reever et al	2006	Alzheimer's	√	√	√	√	yes	ACT/CG/CR
Gitlin, Winter et al	2003	Alzheimer's		√	√	√	yes	ACT/CG/CR
Gitlin, Winter, Burke et al	2008	Alzheimer's	√	√	√			ACT/CG
Given, Given, Sikorskii et al	2006	Cancer	√	√	√			ACT/CG
Gluekhauf, Sharma et al	2007	Dementia		√	√			ACT/CG
Gonyea, O'Connor et al	2006	Alzheimer's				√		CR
Grant, Elliot, Weaver et al	2002	Stroke		√	√			ACT/CG
Grant, McKibbin et al	2004	Dementia			√			CG
Haley, Bergman et al	2008	Dementia			√			CG
Haley, Gitlin et al.	2004	Alzheimer's	√	√	√			ACT/CG
Hartke, King et al	2003	Stroke				√		CR
Hazel, McDonnell et al	2004	Schizophrenia	√			√		CR
Hendrix, Abernathy et al	2009	Cancer		√	√			ACT/CG
Hepburn, Lewis et al	2003	Dementia		√	√	√	yes	ACT/CG/CR
Hepburn, Lewis et al	2007	Dementia	√	√	√			ACT/CG
Hepburn, Lewis, et al	2005	Alzheimer's	√	√	√	√	yes	ACT/CG/CR
Hepburn, Tornatore, et al	2001	Dementia	√		√	√		CG/CR
Hilgeman, Burgio, et al	2007	Alzheimer's	√		√	√		CG/CR
Holland, Courier et al	2009	Dementia	√		√			CG
Huyn-Hohnbaum, Villa et al	2008	Disability	√		√			CG
Jang, Lay et al	2004	Alzheimer's			√			CG
King, Baumann et al	2002	Dementia			√			CG
King, Hartke et al	2007	Stroke	√	√	√	√	yes	ACT/CG/CR
Kopelowicz, Zarate et al	2003	Schizophrenia			√	√		CG/CR
Korn, Logsdon, et al	2009	Dementia			√	√		CG/CR
Kuhn, Deleon et al	2001	Alzheimer's	√	√	√	√	yes	ACT/CG/CR
Kuhn, Fulton et al	2004	Alzheimer's			√	√		CG/CR
Kurtz, Kurtz et al	2005	Cancer	√		√	√		CG/CR
Kwak, Salmon et al	2007	End-of-life	√		√	√		CG/CR
Lenz, Perkins et al	2000	Heart	√		√	√		CG/CR
Leutz, Capitman, et al	2002	Multimorbidity		√	√	√	yes	ACT/CG/CR
Logsdon, McCurry, et al	2006	Dementia			√	√		CG/CR
MacKenzie, Wiprzycka et al	2007	Multimorbidity			√	√		CG/CR
Mahoney, Mutschler et al	2008	Dementia	√	√	√	√	yes	ACT/CG/CR
Mahoney, Tarlow et al	2003	Dementia	√		√	√		CG/CR
Martin-Cook, David et al	2005	Dementia		√	√	√	yes	ACT/CG/CR
McCurry, Gibbons et al	2003	Alzheimer's			√	√		CG/CR
McCurry, Gibbons et al	2005	Alzheimer's			√	√		CG/CR
McGinnis, Schulz et al	2006	Dementia	√		√	√		CG/CR
McMillan, Small et al	2006	Cancer	√	√	√	√	yes	ACT/CG/CR
McMillan, Small et al	2007	Cancer	√			√		CR
Mittelman, Haley et al	2006	Alzheimer's				√		CR
Mittelman, Roth et al	2007	Alzheimer's			√			CG
Mittelman, Roth et al (J Psych)	2004	Alzheimer's			√	√		CG/CR
Mittelman, Roth et al (J Geron)	2004	Alzheimer's	√			√		CR
Nichols, Chang et al	2008	Dementia				√		CR
Northouse, Kershaw et al	2005	Cancer	√	√	√	√	yes	ACT/CG/CR
Ostwald, Hepburn et al	2003	Dementia	√		√			CG
Pasacreta, Barg et al	2000	Cancer		√	√			ACT/CG
Phillips	2008	Dementia	√		√			CG
Pillemer and Suitor	2002	Alzheimer's			√			CG
Powers	2006	Multimorbidity		√				ACT
Quayhagen, Corbeil et al	2000	Dementia				√		CR
Rabinowitz, Mausbach et al	2006	Dementia		√	√			ACT/CG
Rabinowitz, Mausbach, et al	2007	Dementia	√	√	√			ACT/CG
Rexilius, Mundt et al	2002	Stem cell trans			√			CG
Rivera, Elliot et al	2008	Traumatic Brain Injury	√	√	√			ACT/CG
Rose, Radciewicz et al	2008	Cancer	√		√			CG
Rose, Taylor et al	2009	Dementia	√	√	√			ACT/CG
Roth, Mittleman et al	2005	Alzheimer's		√	√	√	yes	ACT/CG/CR
Schulz, Czaja et al	2009	Spinal cord	√		√	√		CG/CR
Schwarz, Mion et al	2008	Stroke		√	√	√	yes	ACT/CG/CR
Smith, Toseland et al	2006	Frailty		√	√			ACT/CG
Stern, d"Ambrosio et al	2008	Driving Cessation		√	√			ACT/CG
Teri, Gibbons et al	2003	Alzheimer's	√			√		CR
Teri, McCurry et al	2005	Alzheimer's		√	√	√	yes	ACT/CG/CR
Tompkins, Bell, 2009	2009	Dementia			√	√		CG/CR
Toseland, Smith et al	2006	Alzheimer's				√		CR
Tremont, Davis et al	2008	Dementia	√	√	√	√	yes	ACT/CG/CR
Vickrey, Mittman et al	2006	Dementia		√	√	√	yes	ACT/CG/CR
Waelde, Thompson et al	2004	Dementia		√	√	√	yes	ACT/CG/CR
Walsh, Martin et al	2004	Cancer			√	√		CG/CR
Walsh, Radcliff et al	2007	Cancer	√		√	√		CG/CR
Walsh, Schmidt et al	2003	Cancer	√	√	√	√	yes	ACT/CG/CR
Weuve, Boult et al	2000	High risk			√	√		CG/CR
Winter, Gitlin et al	2007	Dementia			√	√		CG/CR
Wolff, Rand-Giovannetti et al	2009	End-of-life	√		√	√		CG/CR
Won, Fitts et al	2008	Disability	√		√	√		CG/CR

Note: 1. ACT = caregiver activities; CG = caregiver outcomes; CR = Care recipient outcomes.

### Caregiving Activities

**Figure 4:**
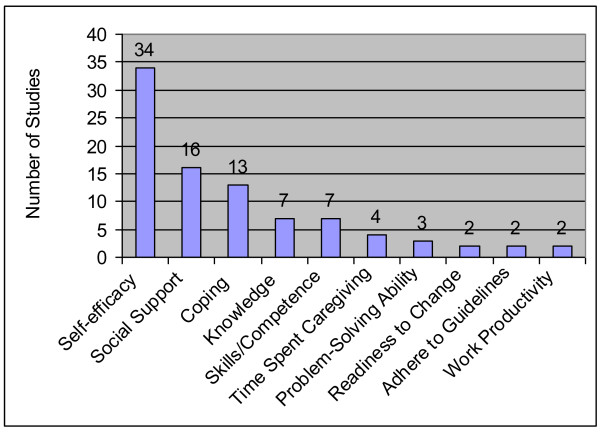
**Top Ten Caregiver Activities**.

Fifty-five studies evaluated changes in caregiving activities, that is, clinical and knowledge, psychological, support seeking, and quantity of caregiving. The ten most frequently assessed caregiving activities appear in Figure 4; the full list of caregiving activities appears in Table 2.

**Table 2:** Caregiving Activities

Construct	Subcategories	# of Studies
- Clinical Skills and Knowledge	Caregiver skills/competence	7
	Knowledge	7
	Problem solving ability	3
	Adhere to care guidelines	2
	Decision-making skills	1
		
- Psychological Skills	Self-efficacy	34
	Coping	13
	Readiness to Change	2
	Appraisal of Illness	1
		
- Support Seeking	Social Support	16
	Intention to Get Support	1
	Attitude toward healthcare for care recipient	1
	Desire to institutionalize care recipient	1
		
- Quantity of Caregiving	Time spent caregiving	4
	Work productivity (absences)	2

#### Clinical and Knowledge

Seven studies focused on improving caregiver's knowledge of disease or expected clinical course [10-13], knowledge about benefits of exercise [14], or knowledge of services [15]. Other clinical aspects of caregiving activities included measures of caregiver skills or competence (7 studies), problem-solving ability (3 studies), adherence to care guidelines (2 studies), and decision-making skills (1 study).

#### Psychological

Self-efficacy was the most commonly assessed psychological skill, with more studies focusing on a caregiver's self-efficacy to perform tasks for the care recipient (28 studies) than on self-efficacy to care for oneself (6 studies). Coping (13 studies) was concerned with dealing with the caregiving role or with the care recipient's health decline. Readiness to change was rarely assessed (3 studies).

#### Support Seeking

Receipt or assessment of social support was the most commonly assessed outcome (16 studies) in this category and included satisfaction with social support and the size and extent of the social network. Other support seeking activities examined included the caregiver's intention to get support (1 study), attitude toward seeking healthcare for the care recipient (1 study), and the desire to institutionalize the care recipient (1 study).

#### Quantity of Caregiving

Only five studies evaluated how an intervention affected quantity of caregiving. Measures of quantity included number of tasks performed [16,17], symptoms managed [18], and days in a week spent caregiving [19].

### Caregiver Outcomes

**Figure 5:**
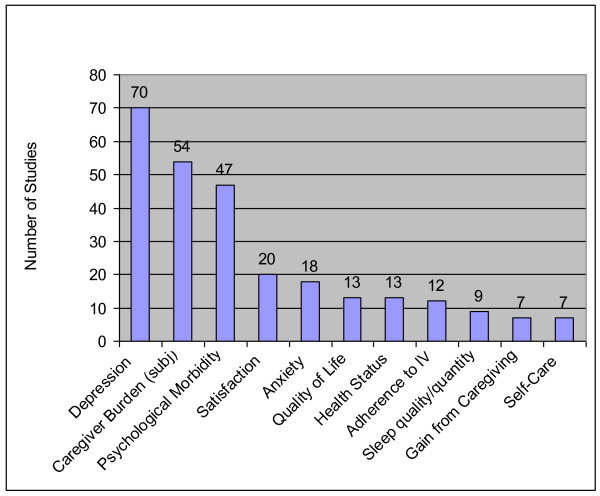
**Top Ten Caregiver Outcomes**. IV =  Intervention; subj = subjective.

Across the 121 studies, 312 caregiver outcomes were reported in the four categories. The ten most frequently assessed caregiver outcomes appear in Figure 5; the full list of caregiver outcomes appear in Table 3.

**Table 3:** Caregiver outcomes

Construct	Subcategories	# of Studies
Psychological Health		
- Nonsocial	Depression	70
	Caregiver Burden	54
	Psychological Morbidity^ii^	47
	Anxiety	18
	Quality of Life	13
	Threat	1
	Self-esteem	1
	Religiosity	1
		
- Social Functioning	Family Role Function	5
	Social Dysfunction	1
	Social Integration	1
		
- Caregiving related	Satisfaction^iii^	20
	Gain from Caregiving	7
	Beliefs about caregiving	2
	% of time spent discussing	1
	family vs. personal issues	
	Role Captivity	1
	Comfort with caregiving	1
	Attribute for negative care recipient behavior	1
	Concern for Care Recipient	1
	Worker morale of caregiver	1
	Closure	1
	Life changes	1
	Tradition	1
	Uncertainty	1
Physical Health		
	Health Status	13
	Sleep quality/quantity	9
	Physiological emotional stress	3
	Somatic symptoms	2
	Fatigue	1
	Immune Function	1
	Blood Pressure/Heart rate	1
	Blood pressure	1
Physical Health		
-Self-Care	Self-Care	7
	Exercise	6
	Dietary Intake	1
	Weight	1
Utilization		
	Use of health care for self	1
Economic		
	Financial status	1
Aspects of the Intervention		
	Adherence to intervention	12
	Benefits of participating in intervention	1

#### Psychological health outcomes

Psychological health outcomes comprised 80% of all caregiver outcomes studied. The most common nonsocial outcomes were depression (70 studies), caregiver subjective burden (54 studies), and psychological morbidity (47 studies). Anxiety (18 studies) and quality of life (13 studies) were also commonly reported (Figure 5).

Another aspect of psychological function, family role function, was examined in five studies. Examples include the caregiver's perception of loss of self and of the relationship with the care recipient due to the disease [20] and family conflicts [21]. Social dysfunction, such as avoiding social situations [22] and social integration of the caregiver in the community [23], were measured in one study each.

Of the caregiving-related psychological outcomes, caregiver satisfaction was a commonly reported outcome (assessed in 20 studies). Closely related, gains or positive aspects of caregiving were measured in 7 studies. The remaining outcomes in this category were assessed only once or twice, including role captivity, comfort with caregiving, or concern for the care recipient (Table 3).

#### Physical health

The most common physical health outcomes included self-rated health (13 studies) and the quality and quantity of sleep (9 studies) (Figure 5). Several studies focused on physiological responses to being a caregiver, either through change in immune function, change in blood pressure, or change in somatic symptoms (Table 3). One physical health outcome related to self-care, which included taking breaks from caregiving or doing things for oneself (7 studies).

#### Utilization

Only one study examined changes in caregiver health care utilization: Haley and colleagues examined whether there was differential psychotropic medication uses among African American Caregivers compared to White caregivers [24].

#### Economic Status

Only one study considered the effects of an intervention on a caregiver's financial status. Specifically, Dellasega and Zerbe [25] evaluated how their intervention affected financial status indirectly by adding an item on 'time missed from work related to caregiving activities.' No study considered the cost of time missed from work, the cost of a caregiver's own health care utilization or the cost of seeking more supportive care.

### Care Recipient Outcomes

**Figure 6:**
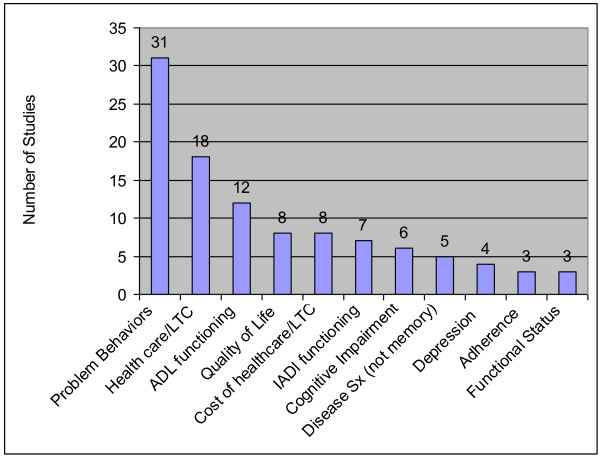
**Top Ten Care Recipient Outcomes**. LTC = long-term care; SX = symptoms; ADL = Activities of Daily Living; IADL = Instrumental Activity of Daily Living.

There were 122 care recipient outcomes in the four categories, the vast majority of which fell into two categories: physical health and health care utilization. The ten most commonly assessed care recipient outcomes appear in Figure 6; the full list of care recipient outcomes, including their frequencies, appear in Table 4.

**Table 4:** Care Recipient Outcomes

Construct	Subcategories	# of Studies
Disease Management	Medical/Medication Adherence	3
	Coping	1
	Appraisal of Illness	1
	Knowledge of Medication/skills	1
	Management skills	1
Psychological Health		
- nonsocial	Quality of Life	8
	Depression	4
	Anxiety	1
	Mental health status	1
	Mood	1
	Hopelessness	1
Physical health		
-physical manifestation of disease	Problem Behaviors	31
	Activity of Daily Living functioning	12
	Instrumental Activities of Daily Living functioning	7
	Cognitive Impairment	6
	Disease Symptoms (other than memory)^iv^	5
	Functional Status	3
	Survival	2
	Aggression	1
	Social Functioning	1
	Family Functioning	1
	Dementia severity	1
	Health Status	1
Utilization		
	Health care/long-term care utilization^v^	18
	Days in the community	1
Economic		
	Cost of healthcare or long-term care	8
Aspects of the Intervention		
	Satisfaction	1

^ii^Including stress, affect or mood, anger, loneliness, general wellbeing, hopelessness, or grief.

^iii^With the intervention, caregiver-care recipient relationships, life, and recipient's care.

^iv^Post-surgical symptoms, pain, and dyspnea.

^v^Home health care, respite care, adult day care use, days in the nursing home before death, any nursing home use, transportation to medical care, emergency department use, primary care, and hospitalization.

#### Psychological Health

Studies evaluating psychological health effects of interventions on care recipients focused on nonsocial psychological outcomes, including quality of life (8 studies), change in depressive symptoms (4 studies), or psychological health measures such as mood or helplessness (4 studies).

#### Physical Health

The most commonly assessed physical manifestation of disease was problem behaviors (31 studies; Figure 6). Other common outcomes in this sub-category were ADL functioning (12 studies), IADL functioning (7 studies), and cognitive impairment (6 studies). Non-memory related disease symptoms in non-dementia studies were also measured (5 studies).

Also a part of this category, care recipients engage in their own disease management. Three studies evaluated care recipients' performance in adhering to appointments or adherence. The remaining outcomes in this category were assessment of the care recipient's skill at managing and coping with his or her own care.

#### Utilization

Health care utilization was the second most commonly assessed outcome for care recipients (Figure 6). Four studies assessed changes in community-based long-term care, and 5 studies assessed changes in nursing home use. Others assessed days in the community before entry, days in the nursing home before death, any nursing home use transportation to medical care, emergency department use, primary care, and hospitalization.

#### Economic Status

Eight studies examined whether there was a change of economic status resulting from a change in costs of health care services consumed. These studies examined total, medication, hospital, outpatient, and emergency room costs. We found no examples of caregiver interventions that considered care recipient economic status beyond health care costs.

## Discussion

This paper provides an organizing framework for developing and evaluating interventions and programs that target informal caregivers. Initially developed by our study team and then refined based upon a structured literature review, the organizing framework explicitly describes and categorizes important caregiving activities and outcomes of both caregivers and care recipients. We assessed the frequency with which components of the framework have been addressed in reports of caregiving intervention studies so that we could identify gaps in the literature that deserve attention in future applications. Our model leads to three recommendations for future researchers, policy makers, agencies, and insurers.

### Recommendation One

#### Caregiver interventions should assess the quantity and/or quality of care provided

Measuring caregiving activities is important to gain a full understanding of an intervention's effect. An intervention may have no effect on caregiver outcomes yet still affect caregiving activities. In such cases, we would be remiss to conclude that the intervention is ineffective. We have two specific suggestions related to the measurement of caregiving activities.

##### Measure and report on quantity of care provided

Without knowing how much time caregivers spend providing care, it is difficult to understand the competing time demands faced by caregivers. More broadly, it is difficult to assess the broad impact and cost-effectiveness of informal caregiving interventions. Notably, measures to capture quantity of care exist, including surveys and diaries, and each have advantages and disadvantages for data collection that the researchers will need to consider during the intervention design phase. Yet virtually all (96%) of the reports we reviewed failed to report the quantity of care supplied.

##### Develop and use measures of caregiving quality

As an effort to better measure caregiving activities, we also recommend that quality of informal care be measured. Our review indicated that there is a dearth of quality measures related to informal care interventions. This may be due to challenges in its measurement. Direct measures (e.g., observation by a social worker) may provide biased estimates of caregiver behaviors due to the Hawthorne effect and substantial respondent burden and cost. We could only find two direct measures of quality [26]; neither was used in any of the reports in our review or has had been used in the broader literature to date. Thus, the first three components of our framework's caregiving activities (clinical, support seeking, and psychological) can be used as proxy measures of quality.

Rather than using caregiving activities as a proxy for quality, researchers need to develop pragmatic, valid measures to assess the quality of informal care. It may be reasonable to use measures that have been developed for other, but related, purposes. For example, home health care quality measures were recently released by the Centers for Medicare and Medicaid Services http://www.medicare.gov/hhcompare/home.asp and could be useful in the context of informal care. Alternatively, one could adapt measures from outcome-based measures of nursing home quality such as acquired pressure sores [27], use of restraints [27,28] or urinary tract infections [29]. Finally, indirect measures of quality of caregiving (e.g., care recipient's receipt of annual flu shots [30]) could also be useful. It is likely that a multi-component measure of caregiver quality is needed to encompass the diverse types of activities provided by caregiver--clinical skills and knowledge, support seeking skills, psychological skills and resources, and quantity of caregiving.

Ideally, a researcher would measure quantity and quality of caregiving activities, because understanding one helps give context to the other. If there is no intent to change quantity of care provided, however, it may be of little interest to researchers to measure quantity on top of quality, but it still may be useful (and relatively easy to do) in order to gauge whether there are unintended consequences from an intervention, such as increasing or decreasing hours of caregiving performed. For this reason, we argue that without measuring both aspects of caregiving, quantity and quality, there is a risk of masking the effects of an intervention. For example, interventions that help caregivers perform clinical tasks more skillfully and efficiently (quality) may reduce the time required to provide care (quantity), and we would be remiss to conclude that decreased quantity indicates a reduction in the quality of caregiving received. If the researcher is interested in measuring only one, then we recommend that quality be the focus, because it is likely to be more directly related to caregiver and care recipient outcomes.

### Recommendation Two

#### Interventions should consider a broader range of caregiver and care recipient outcomes

Because the ultimate goal of health-related interventions is to improve psychological and health outcomes of caregivers and care recipients, the focus of most caregiver intervention studies has been on assessing depressive symptoms, caregiver burden, and nursing home entry. This focus explains why the dominant conceptual model in caregiving research in the past 20 years is the Pearlin Model of Alzheimer's Caregivers' Stress. In the Pearlin model, which focuses on the role of caregiver psychological outcomes, the outcomes of ultimate interest are closely related to stress (depression, anxiety, irascibility, cognitive disturbance, physical health, and yielding of role). Our organizing framework moves beyond the Pearlin model to include caregiver and care recipient activities and outcomes. Considering an expanded view of the critical areas highlighted in our organizing framework will enable researchers to understand more fully the net benefits of informal caregiving interventions for caregivers and their care recipients.

If the only outcomes that caregiver intervention studies consistently and clinically improve are caregiver depressive symptoms and burden [31], then considering the associated costs or savings of alleviating caregiver depression and burden is an important step toward estimating an intervention's full benefit. Specifically, the economic costs associated with health status changes of the caregiver and care recipient, as well as the changes in health care utilization of the caregiver, are important pieces of the outcomes puzzle that are less commonly considered [32]. Studies have neglected, with one or two exceptions, to report effects of an intervention on the caregiver's own health care utilization or the effects on a caregiver's own economic status changes, such as through changes in utilization of primary care (if they seek treatment for stress, strain or depressive symptoms) or medication (for psychotropic medications). Utilization or treatment changes and associated costs were largely missed in the interventions conducted between 2000 and 2009 in the U.S.

Caregiver interventions can have significant economic consequences for the caregiver by encouraging the use of paid services in the home or delaying institutionalization of the care recipient. Importantly, more respite use may decrease current and future economic well being of the caregiver and care recipient if it is costly to purchase such care. On the other hand, if an intervention delays institutionalization of the care recipient, it can improve current and future economic well-being of the caregiver and care recipient. A less direct economic consequence is that caregiver economic status may be altered due to changes in a caregiver's work behavior. If less time is spent caregiving when more respite care is used and more time is spent caregiving if institutionalization is delayed, there could be opposing effects on a caregiver's ability to remain employed.

Finally, interventions can affect economic status of caregivers by affecting a caregiver's out-of-pocket expenditures either through direct medical expenditures (e.g., hiring an aide), indirect medical expenditures (e.g., driving the care recipient to the doctor), or indirect non-medical expenses (e.g., buying prepared meals for the care recipient). Essentially, interventions that affect economic status can have ripple effects by changing the expected value of the estate that will be passed down to a caregiver, by affecting short-term family savings, or simply by affecting the monthly family budget. Depending on how interlinked a caregiver and a care recipient are, the economic effects may be experienced by both individuals in the dyad.

Valuing economic consequences of informal care is difficult because good cost data often are not collected. It is even rarer to perform an economic evaluation of a caregiver intervention study. Only one study examined the cost-effectiveness of the intervention [33] and no study measured quality adjusted life years or QALYs, a standardized utility-based measure of quality of life [34]. Performing cost-effectiveness analysis of interventions would be very helpful for policy purposes and cross-study comparison. Ultimately interventionists have to consider *a priori *what economic consequences may arise from the intervention they are designing and whether they will assess changes in economic status using changes in caregiving quantity, caregiver utilization, work behavior and/or changes in caregiver or care recipient out-of-pocket expenses. If measuring economic outcomes can be easily accommodated by researchers, without adding too much burden to the assessment of measures, it could greatly benefit subsequent economic analyses of caregiver trials.

### Recommendation Three

#### Interventions should consider a common set of caregiver and care recipient outcomes to facilitate comparison across studies and over time

Given that caregiver interventions have resulted in a consistent, albeit modest, improvement on caregiver burden and depression in dementia caregivers [31,35-37], we recommend retaining these two important caregiver outcomes in all studies. Knowing the outcomes of these two psychological measures will facilitate comparisons across studies and over time and allow for more robust syntheses of results. For care recipients, it is not easy to identify one or two common outcomes measured across the majority of studies, likely due to the overwhelming focus on caregiver outcomes and the diverse diagnoses of care recipients in the sample. The most common outcome for care recipients was problem behaviors (31 studies), which is relevant to dementia caregiving but not other types of caregiving. For dementia trials, it is sensible to measure problem behaviors to facilitate comparison. For all caregiver trials, we urge others to adapt a more general measure of care recipient well-being, such as a validated quality of life measure (appearing in 8 studies), in order to allow for better standardization and comparison of care recipients across studies and across diagnoses.

## Conclusion

Our ability to maximize the usefulness of caregiver interventions is limited by the lack of organized framework to define interventions and compare outcomes across studies. The absence of such a framework hinders the field from moving forward despite an explosion in the number of caregiver intervention studies published in the past decade. Interventions and programs have had different goals, mechanisms, and targets, and there has been little harmonization of evaluation across studies [38-40].

Moving beyond the research setting, our organizing framework may be useful for policymakers and practitioners because it will allow them to consider the direct and indirect impact of caregiving to caregivers, care recipients, family members, employers, and broader society [41]. Although our framework focuses on intervention research, it can easily be extended to consider the effects of training on diverse stakeholders. For example, the National Caregiver Support Program has been implemented over the past eight years as a part of the Older Americans Act Amendments (2000). Across all 50 states, different agencies have received funds to offer training and respite services to family caregivers, and evaluations of the program may find the framework useful in assessing the impact on skill and outcomes, by helping to identifying both the processes and important outcomes for consideration. The framework can be used regardless of the perspective of the evaluation (i.e., caregivers, health system, or society), and because it offers standardization, it can allow for ready comparisons to be made across settings.

### Limitations

Our organizational framework was informed by input from experts on caregiving and aging research and a review of U.S. caregiver trial literature from 2000-2010. As such, we may have a biased view about the gaps in measures due to differences in emphasis prior to the year 2000 and/or different emphases in the international literature. For example, initial reviews of the international literature showed more frequent considered of the cost-effectiveness of a program (primarily in the United Kingdom). Therefore, we may be overstating the lack of interest in economic outcomes world-wide. The U.S. focus was deemed necessary because other countries have different policies and cultural contexts that might affect models of informal caregiving. Furthermore, our search terms strategy may have missed key evaluations of studies, but checking the identified articles against articles from systematic reviews in the literature from the 2000s did not reveal additional articles, so we are confident that we have captured most if not all of the pre-eminent caregiver trials in the U.S. published in the 2000s. Another limitation is that we did not focus on the content of the interventions so they are extremely heterogeneous in approaches, doses, and their objectives--all had in common that they were trying to improve caregiver and/or care recipient health and/or functioning.

Lack of standardization and an increasing role for caregivers in society in the coming decade means that it is time to design and evaluate caregiving interventions systematically. Only by doing this, and by considering the specific caregiving activities and health and economic outcomes of both caregivers and care recipients, will we be able to fully consider the net benefits of informal care interventions and the net benefits of informal care more generally. In turn, understanding the net benefits of informal care will help inform evidenced-based policy decisions on how best to allocate scarce resources in the future.

## Competing interests

The authors declare that they have no competing interests.

## Authors' contributions

CHV drafted the original framework and CHV and MW wrote the initial draft. CHV and CIV performed the literature review, data analysis, and revisions to the model. CIV provided key methodological expertise for the review. CHV, CIV and MW all worked on rewriting the draft of the paper. All authors read and approved the final manuscript.

## Pre-publication history

The pre-publication history for this paper can be accessed here:

http://www.biomedcentral.com/1471-2318/11/77/prepub

## Supplementary Material

Additional file 1 **Structured Literature Review References**. 121 individual reports were included in our analysis. The full list of references for these studies appears in this file. Click here for file
