# Supplementary material for: An organizing framework for informal caregiver interventions: detailing caregiving activities and caregiver and care recipient outcomes to optimize evaluation efforts
Source: BMC Geriatr. 2011 Nov 22;11:77. doi: 10.1186/1471-2318-11-77 (PMC3258201; doi:10.1186/1471-2318-11-77)
Supplement: Additional file 1 — Structured Literature Review References. 121 individual reports were included in our analysis. The full list of references for these studies appears in this file. [file 1471-2318-11-77-S1.DOC]

**Structured Literature Review References (N=121)**

Akkerman, R. L., & Ostwald, S. K. (2004). Reducing anxiety in Alzheimer's disease family caregivers: The effectiveness of a nine-week cognitive-behavioral intervention. *American Journal of Alzheimer's Disease and Other Dementias, 19*(2), 117-123.

Albert, S. M., Im, A., Brenner, L., Smith, M., & Waxman, R. (2002). Effect of a social work liaison program on family caregivers to people with brain injury. *Journal of Head Trauma Rehabilitation, 17*(2), 175-189.

Bakas, T., Farran, C. J., Austin, J. K., Given, B. A., Johnson, E. A., Williams, L. S., et al. (2009). Stroke caregiver outcomes from the Telephone Assessment and Skill-Building Kit (TASK). *Topics in Stroke Rehabilitation, 16*(2), 105-121.

Bank, A. L., Arguelles, S., Rubert, M., Eisdorfer, C., & Czaja, S. J. (2006). The value of telephone support groups among ethnically diverse caregivers of persons with dementia. *Gerontologist, 46*(1), 134-138.

Beauchamp, N., Irvine, A. B., Seeley, J., & Johnson, B. (2005). Worksite-based internet multimedia program for family caregivers of persons with dementia. *Gerontologist, 45*(6), 793-801.

Belle, S. H., Burgio, L., Burns, R., Coon, D., Czaja, S. J., Gallagher-Thompson, D., et al. (2006). Enhancing the quality of life of dementia caregivers from different ethnic or racial groups: a randomized, controlled trial.[summary for patients in Ann Intern Med. 2006 Nov 21;145(10):I39; PMID: 17116914]. *Annals of Internal Medicine, 145*(10), 727-738.

Boerner, K., Schulz, R., & Horowitz, A. (2004). Positive Aspects of Caregiving and Adaptation to Bereavement. *Psychology and Aging, 19*(4), 668-675.

Bourgeois, M., Schulz, R., Burgio, L., & Beach, S. (2002). Skills training for spouses of patients with Alzheimer’s disease: Outcomes of an intervention study. *Journal of Clinical Geropsychology, 53*, 53-73.

Burgio, L., Stevens, A., Guy, D., Roth, D. L., & Haley, W. E. (2003). Impact of two psychosocial interventions on white and African American family caregivers of individuals with dementia. *Gerontologist, 43*(4), 568-579.

Burns, R., Nichols, L., Martindale Adams, J., Graney, M., & Lummus, A. (2003). Primary care interventions for dementia caregivers: 2-year outcomes from the reach study. *Gerontologist, 43*(4), 173-181.

Callahan, C. M., Boustani, M. A., Unverzagt, F. W., Austrom, M. G., Damush, T. M., Perkins, A. J., et al. (2006). Effectiveness of collaborative care for older adults with Alzheimer disease in primary care: a randomized controlled trial. *JAMA, 295*(18), 2148-2157.

Carnevale, G. J., Anselmi, V., Busichio, K., & Millis, S. R. (2002). Changes in ratings of caregiver burden following a community-based behavior management program for persons with traumatic brain injury. *J Head Trauma Rehabil, 17*(2), 83-95.

Carter, P. A. (2006). A brief behavioral sleep intervention for family caregivers of persons with cancer. *Cancer Nursing, 29*(2), 95-103.

Castro, C., Wilcox, S., O'Sullivan, P., Baumann, K., & King, A. (2002). An exercise program for women who are caring for relatives with dementia. *Psychosomatic Medicine, 64*, 458-468.

Chee, Y. K., Gitlin, L. N., Dennis, M. P., & Hauck, W. W. (2007). Predictors of adherence to a skill-building intervention in dementia caregivers. *Journals of Gerontology Series A-Biological Sciences & Medical Sciences, 62*(6), 673-678.

Clark, M. C., & Lester, J. (2000). The effect of video-based interventions on self-care. *West J Nurs Res, 22*(8), 895-911.

Clark, M. M., Rummans, T. A., Sloan, J. A., Jensen, A., Atherton, P. J., Frost, M. H., et al. (2006). Quality of life of caregivers of patients with advanced-stage cancer. *American Journal of Hospice & Palliative Medicine, 23*(3), 185-191.

Connell, C. M., & Janevic, M. R. (2009). Effects of a telephone-based exercise intervention for dementia caregiving wives: a randomized controlled trial. *Journal of Applied Gerontology, 28*(2), 171-194.

Coon, D. W., Thompson, L., Steffen, A., Sorocco, K., & Gallagher-Thompson, D. (2003). Anger and depression management: psychoeducational skill training interventions for women caregivers of a relative with dementia. *Gerontologist, 43*(5), 678-689.

Corcoran, M. A., & Gitlin, L. N. (2001). Family caregiver acceptance and use of environmental strategies provided in an occupational therapy intervention. *Physical & Occupational Therapy in Geriatrics, 19*(1), 1-20.

Dellasega, C., & Zerbe, T. M. (2002). Caregivers of frail rural older adults. Effects of an advanced practice nursing intervention. *Journal of Gerontological Nursing, 28*(10), 40-49.

Devor, M., & Renvall, M. (2008). An educational intervention to support caregivers of elders with dementia. *American Journal of Alzheimer's Disease & Other Dementias, 23*(3), 233-241.

Dew, M. A., Goycoolea, J. M., Harris, R. C., Lee, A., Zomak, R., Dunbar-Jacob, J., et al. (2004). An internet-based intervention to improve psychosocial outcomes in heart transplant recipients and family caregivers: development and evaluation. *Journal of Heart & Lung Transplantation, 23*(6), 745-758.

Drentea, P., Clay, O. J., Roth, D. L., & Mittelman, M. S. (2006). Predictors of improvement in social support: Five-year effects of a structured intervention for caregivers of spouses with Alzheimer's disease. *Soc Sci Med, 63*(4), 957-967.

Eisdorfer, C., Czaja, S. J., Loewenstein, D. A., Rubert, M. P., Arguelles, S., Mitrani, V. B., et al. (2003). The effect of a family therapy and technology-based intervention on caregiver depression. *Gerontologist, 43*(4), 521-531.

Elliott, T. R., & Berry, J. W. (2009). Brief problem-solving training for family caregivers of persons with recent-onset spinal cord injuries: a randomized controlled trial. *Journal of Clinical Psychology, 65*(4), 406-422.

Elliott, T. R., Brossart, D., Berry, J. W., & Fine, P. R. (2008). Problem-solving training via videoconferencing for family caregivers of persons with spinal cord injuries: a randomized controlled trial. *Behaviour Research & Therapy, 46*(11), 1220-1229.

Farran, C. J., Gilley, D. W., McCann, J. J., Bienias, J. L., Lindeman, D. A., & Evans, D. A. (2004). Psychosocial interventions to reduce depressive symptoms of dementia caregivers: a randomized clinical trial comparing two approaches. *Journal of Mental Health & Aging, 10*(4), 337-350.

Farran, C. J., Gilley, D. W., McCann, J. J., Bienias, J. L., Lindeman, D. A., & Evans, D. A. (2007). Efficacy of Behavioral Interventions for Dementia Caregivers. *West J Nurs Res, 29*(8), 944-960.

Farran, C. J., Staffileno, B. A., Gilley, D. W., McCann, J. J., Yan, L., Castro, C. M., et al. (2008). A lifestyle physical activity intervention for caregivers of persons with Alzheimer's disease. *American Journal of Alzheimer's Disease & Other Dementias, 23*(2), 132-142.

Finkel, S., Czaja, S. J., Schulz, R., Martinovich, Z., Harris, C., Pezzuto, D., et al. (2007). E-care: a telecommunications technology intervention for family caregivers of dementia patients. *American Journal of Geriatric Psychiatry, 15*(5), 443-448.

Fortinsky, R. H., Kulldorff, M., Kleppinger, A., Kenyon-Pesce, L., Fortinsky, R. H., Kulldorff, M., et al. (2009). Dementia care consultation for family caregivers: collaborative model linking an Alzheimer's association chapter with primary care physicians. *Aging & Mental Health, 13*(2), 162-170.

Gallagher-Thompson, D., Coon, D. W., Solano, N., Ambler, C., Rabinowitz, Y., & Thompson, L. W. (2003). Change in indices of distress among Latino and Anglo female caregivers of elderly relatives with dementia: site-specific results from the REACH national collaborative study. *Gerontologist, 43*(4), 580-591.

Gallagher-Thompson, D., Gray, H. L., Tang, P. C., Pu, C. Y., Leung, L. Y., Wang, P. C., et al. (2007). Impact of in-home behavioral management versus telephone support to reduce depressive symptoms and perceived stress in Chinese caregivers: results of a pilot study. *American Journal of Geriatric Psychiatry, 15*(5), 425-434.

Gallagher-Thompson, D., Lovett, S., Rose, J., McKibbin, C., Coon, D., Futterman, A., et al. (2000). Impact of phsychoeducational interventions on distressed family caregivers. *Journal of CClinical Geropsychology, 6*(2), 91-110.

Gant, J. R., Steffen, A. M., & Lauderdale, S. A. (2007). Comparative outcomes of two distance-based interventions for male caregivers of family members with dementia. *American Journal of Alzheimer's Disease & Other Dementias, 22*(2), 120-128.

Garand, L., Buckwalter, K. C., Lubaroff, L., Tripp-Reimer, T., Frantz, R., & Ansley, T. (2002). A Pilot Study of Immune and Mood Outcomes of a Community-Based Intervention for Dementia Caregivers: The PLST Intervention. *Archives of Psychiatric Nursing, 16*(4), 156-167.

Gaugler, J. E., Roth, D. L., Haley, W. E., & Mittelman, M. S. (2008). Can counseling and support reduce burden and depressive symptoms in caregivers of people with Alzheimer's disease during the transition to institutionalization? Results from the New York University caregiver intervention study. *J Am Geriatr Soc, 56*(3), 421-428.

Gerdner, L. A., Buckwalter, K. C., & Reed, D. (2002). Impact of a psychoeducational intervention on caregiver response to behavioral problems. *Nurs Res, 51*(6), 363-374.

Gitlin, L., Corcoran, M., Winter, L., Boyce, A., & Hauck, W. W. (2001). A randomized, controlled trial of a home environmental intervention: effect on efficacy and upset in caregivers and on daily function of persons with dementia. *Gerontologist, 41*(1), 4-14.

Gitlin, L. N., Belle, S. H., Burgio, L. D., Czaja, S. J., Mahoney, D., Gallagher-Thompson, D., et al. (2003). Effect of multicomponent interventions on caregiver burden and depression: the REACH multisite initiative at 6-month follow-up. *Psychol Aging, 18*(3), 361-374.

Gitlin, L. N., Hauck, W. W., Dennis, M. P., Winter, L., Gitlin, L. N., Hauck, W. W., et al. (2005). Maintenance of effects of the home environmental skill-building program for family caregivers and individuals with Alzheimer's disease and related disorders. *Journals of Gerontology Series A-Biological Sciences & Medical Sciences, 60*(3), 368-374.

Gitlin, L. N., Hauck, W. W., Winter, L., Dennis, M. P., & Schulz, R. (2006). Effect of an in-home occupational and physical therapy intervention on reducing mortality in functionally vulnerable older people: preliminary findings. *J Am Geriatr Soc, 54*(6), 950-955.

Gitlin, L. N., Reever, K., Dennis, M. P., Mathieu, E., Hauck, W. W., Gitlin, L. N., et al. (2006). Enhancing quality of life of families who use adult day services: Short- and long-term effects of the adult day services plus program. *Gerontologist, 46*(5), 630-639.

Gitlin, L. N., Winter, L., Burke, J., Chernett, N., Dennis, M. P., & Hauck, W. W. (2008). Tailored activities to manage neuropsychiatric behaviors in persons with dementia and reduce caregiver burden: a randomized pilot study. *Am J Geriatr Psychiatry, 16*(3), 229-239.

Gitlin, L. N., Winter, L., Dennis, M. P., & Hauck, W. W. (2006). Assessing perceived change in the well-being of family caregivers: psychometric properties of the Perceived Change Index and response patterns. *Am J Alzheimers Dis Other Demen, 21*(5), 304-311.

Given, B., Given, C. W., Sikorskii, A., Jeon, S., Sherwood, P., Rahbar, M., et al. (2006). The impact of providing symptom management assistance on caregiver reaction: results of a randomized trial. *Journal of Pain & Symptom Management, 32*(5), 433-443.

Glueckauf, R. L., Sharma, D., Davis, W. S., Byrd, V., Stine, C., Jeffers, S. B., et al. (2007). Telephone-based cognitive-behavioral intervention for distressed rural dementia caregivers: initial findings. *Clinical Gerontologist, 31*(1), 21-41.

Gonyea, J. G., O'Connor, M. K., & Boyle, P. A. (2006). Project CARE: A Randomized Controlled Trial of a Behavioral Intervention Group for Alzheimer's Disease Caregivers. *The Gerontologist, 46*(6), 827-832.

Grant, I., McKibbin, C. L., Taylor, M. J., Mills, P., Dimsdale, J., Ziegler, M., et al. (2003). In-home respite intervention reduces plasma epinephrine in stressed Alzheimer caregivers. *American Journal of Geriatric Psychiatry, 11*(1), 62-72.

Grant, J. S., Elliott, T. R., Weaver, M., Bartolucci, A. A., Giger, J. N., Grant, J. S., et al. (2002). Telephone intervention with family caregivers of stroke survivors after rehabilitation.[see comment]. *Stroke, 33*(8), 2060-2065.

Haley, W. E., Bergman, E. J., Roth, D. L., McVie, T., Gaugler, J. E., Mittelman, M. S., et al. (2008). Long-term effects of bereavement and caregiver intervention on dementia caregiver depressive symptoms. *Gerontologist, 48*(6), 732-740.

Haley, W. E., Gitlin, L. N., Wisniewski, S. R., Mahoney, D. F., Coon, D. W., Winter, L., et al. (2004). Well-being, appraisal, and coping in African-American and Caucasian dementia caregivers: findings from the REACH study. *Aging Ment Health, 8*(4), 316-329.

Hartke, R. J., & King, R. B. (2003). Telephone Group Intervention for Older Stroke Caregivers. *Topics in Stroke Rehabilitation, 9*(4), 65-81.

Hazel, N. A., McDonell, M. G., Short, R. A., Berry, C. M., Voss, W. D., Rodgers, M. L., et al. (2004). Impact of multiple-family groups for outpatients with schizophrenia on caregivers' distress and resources. *Psychiatr Serv, 55*(1), 35-41.

Hendrix, C. C., Abernethy, A., Sloane, R., Misuraca, J., & Moore, J. (2009). A pilot study on the influence of an individualized and experiential training on cancer caregiver's self-efficacy in home care and symptom management. *Home Healthcare Nurse, 27*(5), 271-278.

Hepburn, K., Lewis, M., Tornatore, J., Sherman, C. W., & Bremer, K. L. (2007). The Savvy Caregiver program: the demonstrated effectiveness of a transportable dementia caregiver psychoeducation program. *Journal of Gerontological Nursing, 33*(3), 30-36.

Hepburn, K. W., Lewis, M., Narayan, S., Center, B., Tornatore, J., Bremer, K. L., et al. (2005). Partners in caregiving: a psychoeducation program affecting dementia family caregivers' distress and caregiving outlook. *Clinical Gerontologist, 29*(1), 53-69.

Hepburn, K. W., Lewis, M., Sherman, C. W., & Tornatore, J. (2003). The savvy caregiver program: developing and testing a transportable dementia family caregiver training program. *Gerontologist, 43*(6), 908-915.

Hepburn, K. W., Tornatore, J., Center, B., & Ostwald, S. W. (2001). Dementia family caregiver training: affecting beliefs about caregiving and caregiver outcomes.[see comment]. *Journal of the American Geriatrics Society, 49*(4), 450-457.

Hilgeman, M. M., Allen, R. S., DeCoster, J., Burgio, L. D., Hilgeman, M. M., Allen, R. S., et al. (2007). Positive aspects of caregiving as a moderator of treatment outcome over 12 months. *Psychology & Aging, 22*(2), 361-371.

Holland, J. M., Currier, J. M., & Gallagher-Thompson, D. (2009). Outcomes from the Resources for Enhancing Alzheimer's Caregiver Health (REACH) program for bereaved caregivers. *Psychology & Aging, 24*(1), 190-202.

Huynh-Hohnbaum, A. L., Villa, V. M., Aranda, M. P., & Lambrinos, J. (2008). Evaluating a multicomponent caregiver intervention. *Home Health Care Services Quarterly, 27*(4), 299-325.

Jang, Y., Clay, O. J., Roth, D. L., Haley, W. E., & Mittelman, M. S. (2004). Neuroticism and longitudinal change in caregiver depression: impact of a spouse-caregiver intervention program. *Gerontologist, 44*(3), 311-317.

King, A. C., Baumann, K., O'Sullivan, P., Wilcox, S., Castro, C., King, A. C., et al. (2002). Effects of moderate-intensity exercise on physiological, behavioral, and emotional responses to family caregiving: a randomized controlled trial. *Journals of Gerontology Series A-Biological Sciences & Medical Sciences, 57*(1), M26-36.

King, R. B., Hartke, R. J., & Denby, F. (2007). Problem-solving early intervention: a pilot study of stroke caregivers. *Rehabilitation Nursing, 32*(2), 68-76.

Kopelowicz, A., Zarate, R., Gonzalez Smith, V., Mintz, J., & Liberman, R. P. (2003). Disease management in Latinos with schizophrenia: a family-assisted, skills training approach. *Schizophr Bull, 29*(2), 211-227.

Korn, L., Logsdon, R. G., Polissar, N. L., Gomez-Beloz, A., Waters, T., Ryser, R., et al. (2009). A randomized trial of a CAM therapy for stress reduction in American Indian and Alaskan Native family caregivers. *Gerontologist, 49*(3), 368-377.

Kuhn, D., & Fulton, B. R. (2004). Efficacy of an educational program for relatives of persons in the early stages of Alzheimer's disease. *Journal of Gerontological Social Work, 42*(3/4), 109-130.

Kuhn, D. R., & de Leon, C. F. M. (2001). Evaluating an educational intervention with relatives of persons in the early stages of Alzheimer's disease. *Research on Social Work Practice, 11*(5), 531-548.

Kurtz, M. E., Kurtz, J. C., Given, C. W., & Given, B. (2005). A randomized, controlled trial of a patient/caregiver symptom control intervention: effects on depressive symptomatology of caregivers of cancer patients. *Journal of Pain & Symptom Management, 30*(2), 112-122.

Kwak, J., Salmon, J. R., Acquaviva, K. D., Brandt, K., & Egan, K. A. (2007). Benefits of training family caregivers on experiences of closure during end-of-life care. *Journal of Pain & Symptom Management, 33*(4), 434-445.

Lenz, E. R., & Perkins, S. (2000). Coronary artery bypass graft surgery patients and their family member caregivers: outcomes of a family-focused staged psychoeducational intervention.[see comment]. *Applied Nursing Research, 13*(3), 142-150.

Leutz, W., Capitman, J., Ruwe, M., Ching, V., Flaherty-Robb, M., McKenzie, M., et al. (2002). Caregiver Education and Support: Results of a Multi-Site Pilot in an HMO. *Home Health Care Services Quarterly, 21*(2), 49-73.

Logsdon, R. G., McCurry, S. M., & Teri, L. (2007). Time-limited support groups for individuals with early stage dementia and their care partners: preliminary outcomes from a controlled clinical trial. *Clinical Gerontologist, 30*(2), 5-19.

Mackenzie, C. S., Wiprzycka, U. J., Hasher, L., & Goldstein, D. (2007). Does expressive writing reduce stress and improve health for family caregivers of older adults? *Gerontologist, 47*(3), 296-306.

Mahoney, D. F., Tarlow, B. J., Jones, R. N., Mahoney, D. F., Tarlow, B. J., & Jones, R. N. (2003). Effects of an automated telephone support system on caregiver burden and anxiety: findings from the REACH for TLC intervention study. *Gerontologist, 43*(4), 556-567.

Mahoney, D. M., Mutschler, P. H., Tarlow, B., Liss, E., Mahoney, D. M. F., Mutschler, P. H., et al. (2008). Real world implementation lessons and outcomes from the Worker Interactive Networking (WIN) project: workplace-based online caregiver support and remote monitoring of elders at home. *Telemedicine Journal & E-Health, 14*(3), 224-234.

Martin-Cook, K., Davis, B. A., Hynan, L. S., & Weiner, M. F. (2005). A randomized, controlled study of an Alzheimer's caregiver skills training program. *American Journal of Alzheimer's Disease and Other Dementias, 20*(4), 204-210.

McCurry, S. M., Gibbons, L. E., Logsdon, R. G., Vitiello, M., & Teri, L. (2003). Training caregivers to change the sleep hygiene practices of patients with dementia: the NITE-AD project. *Journal of the American Geriatrics Society, 51*(10), 1455-1460.

McCurry, S. M., Gibbons, L. E., Logsdon, R. G., Vitiello, M. V., & Teri, L. (2005). Nighttime insomnia treatment and education for Alzheimer's disease: a randomized, controlled trial. *Journal of the American Geriatrics Society, 53*(5), 793-802.

McGinnis, K. A., Schulz, R., Stone, R. A., Klinger, J., & Mercurio, R. (2006). Concordance of race or ethnicity of interventionists and caregivers of dementia patients: relationship to attrition and treatment outcomes in the REACH study. *Gerontologist, 46*(4), 449-455.

McMillan, S. C., & Small, B. J. (2007). Using the COPE intervention for family caregivers to improve symptoms of hospice homecare patients: a clinical trial. *Oncology Nursing Forum Online, 34*(2), 313-321.

McMillan, S. C., Small, B. J., Weitzner, M., Schonwetter, R., Tittle, M., Moody, L., et al. (2006). Impact of coping skills intervention with family caregivers of hospice patients with cancer: a randomized clinical trial. *Cancer, 106*(1), 214-222.

Mittelman, M. S., Haley, W. E., Clay, O. J., & Roth, D. L. (2006). Improving caregiver well-being delays nursing home placement of patients with Alzheimer disease. *Neurology, 67*(9), 1592-1599.

Mittelman, M. S., Roth, D. L., Clay, O. J., & Haley, W. E. (2007). Preserving health of Alzheimer caregivers: impact of a spouse caregiver intervention. *Am J Geriatr Psychiatry, 15*(9), 780-789.

Mittelman, M. S., Roth, D. L., Coon, D. W., Haley, W. E., Mittelman, M. S., Roth, D. L., et al. (2004). Sustained benefit of supportive intervention for depressive symptoms in caregivers of patients with Alzheimer's disease. *American Journal of Psychiatry, 161*(5), 850-856.

Mittelman, M. S., Roth, D. L., Haley, W. E., & Zarit, S. H. (2004). Effects of a caregiver intervention on negative caregiver appraisals of behavior problems in patients with Alzheimer's disease: results of a randomized trial. *J Gerontol B Psychol Sci Soc Sci, 59*(1), P27-34.

Nichols, L. O., Chang, C., Lummus, A., Burns, R., Martindale-Adams, J., Graney, M. J., et al. (2008). The cost-effectiveness of a behavior intervention with caregivers of patients with Alzheimer's disease. *J Am Geriatr Soc, 56*(3), 413-420.

Northouse, L., Kershaw, T., Mood, D., & Schafenacker, A. (2005). Effects of a family intervention on the quality of life of women with recurrent breast cancer and their family caregivers. *Psycho-Oncology, 14*(6), 478-491.

Ostwald, S. K., Hepburn, K. W., Burns, T., Ostwald, S. K., Hepburn, K. W., & Burns, T. (2003). Training family caregivers of patients with dementia. A structured workshop approach. *Journal of Gerontological Nursing, 29*(1), 37-44; quiz 55-36.

Pasacreta, J. V., Barg, F., Nuamah, I., McCorkle, R., Pasacreta, J. V., Barg, F., et al. (2000). Participant characteristics before and 4 months after attendance at a family caregiver cancer education program. *Cancer Nursing, 23*(4), 295-303.

Phillips, L. R., (2008). Abuse of aging caregivers: test of a nursing intervention. *Advances in Nursing Science, 31*(2), 164-181.

Pillemer, K., & Suitor, J. J. (2002). Peer support for Alzheimer's caregivers: is it enough to make a difference? *Research on Aging, 24*(2), 171-192.

Powers, S. E. (2006). The Family Caregiver Program: design and effectiveness of an education intervention. *Home Healthcare Nurse, 24*(8), 513-516.

Quayhagen, M. P., Quayhagen, M., Corbeil, R. R., Hendrix, R. C., Jackson, J. E., Snyder, L., et al. (2000). Coping with dementia: evaluation of four nonpharmacologic interventions. *Int Psychogeriatr, 12*(2), 249-265.

Rabinowitz, Y. G., Mausbach, B. T., Coon, D. W., Depp, C., Thompson, L. W., & Gallagher-Thompson, D. (2006). The moderating effect of self-efficacy on intervention response in women family caregivers of older adults with dementia. *Am J Geriatr Psychiatry, 14*(8), 642-649.

Rabinowitz, Y. G., Mausbach, B. T., Thompson, L. W., & Gallagher-Thompson, D. (2007). The relationship between self-efficacy and cumulative health risk associated with health behavior patterns in female caregivers of elderly relatives with Alzheimer's dementia. *J Aging Health, 19*(6), 946-964.

Rexilius, S., Mundt, C., Megel, M., & Agrawal, S. (2002). Therapeutic Effects of Massage Therapy and Healing Touch on Caregivers of Patients Undergoing Autologous Hematopoietic Stem Cell Transplant *Oncology Nursing Forum Online, 29*(3), E35-E44.

Rivera, P. A., Elliott, T. R., Berry, J. W., Grant, J. S., Rivera, P. A., Elliott, T. R., et al. (2008). Problem-solving training for family caregivers of persons with traumatic brain injuries: a randomized controlled trial. *Archives of Physical Medicine & Rehabilitation, 89*(5), 931-941.

Rose, J. H., Radziewicz, R., Bowmans, K. F., & O'Toole, E. E. (2008). A coping and communication support intervention tailored to older patients diagnosed with late-stage cancer. *Clinical Interventions In Aging, 3*(1), 77-95.

Rose, K. M., Taylor, A. G., & Bourguignon, C. (2009). Effects of cranial electrical stimulation on sleep disturbances, depressive symptoms, and caregiving appraisal in spousal caregivers of persons with Alzheimer's disease. *Applied Nursing Research, 22*(2), 119-125.

Roth, D. L., Mittelman, M. S., Clay, O. J., Madan, A., & Haley, W. E. (2005). Changes in social support as mediators of the impact of a psychosocial intervention for spouse caregivers of persons with Alzheimer's disease. *Psychol Aging, 20*(4), 634-644.

Schulz, R., Czaja, S. J., Lustig, A., Zdaniuk, B., Martire, L. M., Perdomo, D., et al. (2009). Improving the quality of life of caregivers of persons with spinal cord injury: a randomized controlled trial. *Rehabilitation Psychology, 54*(1), 1-15.

Schwarz, K. A., Mion, L. C., Hudock, D., & Litman, G. (2008). Telemonitoring of heart failure patients and their caregivers: a pilot randomized controlled trial. *Progress in Cardiovascular Nursing, 23*(1), 18-26.

Smith, T. L., & Toseland, R. W. (2006). The effectiveness of a telephone support program for caregivers of frail older adults. *Gerontologist, 46*(5), 620-629.

Stern, R. A., D'Ambrosio, L. A., Mohyde, M., Carruth, A., Tracton-Bishop, B., Hunter, J. C., et al. (2008). At the crossroads: development and evaluation of a dementia caregiver group intervention to assist in driving cessation. *Gerontology & Geriatrics Education, 29*(4), 363-382.

Teri, L., Gibbons, L. E., McCurry, S. M., Logsdon, R. G., Buchner, D. M., Barlow, W. E., et al. (2003). Exercise plus behavioral management in patients with Alzheimer disease: a randomized controlled trial. *Jama, 290*(15), 2015-2022.

Teri, L., McCurry, S. M., Logsdon, R., Gibbons, L. E., Teri, L., McCurry, S. M., et al. (2005). Training community consultants to help family members improve dementia care: a randomized controlled trial. *Gerontologist, 45*(6), 802-811.

Tompkins, S. A., & Bell, P. A. (2009). Examination of a psychoeducational intervention and a respite grant in relieving psychosocial stressors associated with being an Alzheimer's caregiver. *Journal of Gerontological Social Work, 52*(2), 89-104.

Toseland, R. W., & Smith, T. L. (2006). The impact of a caregiver health education program on health care costs. *Research on Social Work Practice, 16*(1), 9-19.

Tremont, G., Davis, J. D., Bishop, D. S., & Fortinsky, R. H. (2008). Telephone-delivered psychosocial intervention reduces burden in dementia caregivers. *Dementia: The International Journal of Social Research and Practice, 7*(4), 503-520.

Vickrey, B. G., Mittman, B. S., Connor, K. I., Pearson, M. L., Della Penna, R. D., Ganiats, T. G., et al. (2006). The effect of a disease management intervention on quality and outcomes of dementia care: a randomized, controlled trial.[see comment][summary for patients in Ann Intern Med. 2006 Nov 21;145(10):I31; PMID: 17116913]. *Annals of Internal Medicine, 145*(10), 713-726.

Waelde, L. C., Thompson, L., & Gallagher-Thompson, D. (2004). A pilot study of a yoga and meditation intervention for dementia caregiver stress. *Journal of Clinical Psychology, 60*(6), 677-687.

Walsh, S. M., Martin, S. C., & Schmidt, L. A. (2004). Testing the efficacy of a creative-arts intervention with family caregivers of patients with cancer. *Journal of Nursing Scholarship, 36*(3), 214-219.

Walsh, S. M., Radcliffe, R. S., Castillo, L. C., Kumar, A. M., & Broschard, D. M. (2007). A pilot study to test the effects of art-making classes for family caregivers of patients with cancer. *Oncology Nursing Forum Online, 34*(1), 38.

Walsh, S. M., & Schmidt, L. A. (2003). Telephone support for caregivers of patients with cancer. *Cancer Nurs, 26*(6), 448-453.

Weuve, J. L., Boult, C., & Morishita, L. (2000). The effects of outpatient geriatric evaluation and management on caregiver burden. *Gerontologist, 40*(4), 429-436.

Winter, L., & Gitlin, L. N. (2007). Evaluation of a Telephone-Based Support Group Intervention for Female Caregivers of Community-Dwelling Individuals With Dementia. *American Journal of Alzheimer's Disease and Other Dementias, 21*(6), 391-397.

Wolff, J. L., Rand-Giovannetti, E., Palmer, S., Wegener, S., Reider, L., Frey, K., et al. (2009). Caregiving and chronic care: the guided care program for families and friends. *Journals of Gerontology Series A-Biological Sciences & Medical Sciences, 64*(7), 785-791.

Won, C. W., Fitts, S. S., Favaro, S., Olsen, P., & Phelan, E. A. (2008). Community-based "powerful tools" intervention enhances health of caregivers. *Archives of Gerontology & Geriatrics, 46*(1), 89-100.
